# Subclinical Respiratory Involvement in Children with Inflammatory Bowel Disease: FeNO Elevation in Active Disease

**DOI:** 10.3390/children13050711

**Published:** 2026-05-21

**Authors:** Višnja Tokić Pivac, Sanja Kolaček, Iva Hojsak, Zrinjka Mišak, Oleg Jadrešin, Ivan Pavić

**Affiliations:** 1 Children’s Hospital Zagreb, Klaićeva 16, 10000 Zagreb, Croatia; visnja.tokic@kdb.hr (V.T.P.); sanja.kolacek@gmail.com (S.K.); iva.hojsak@kdb.hr (I.H.); oleg.jadresin@gmail.com (O.J.); 2 School of Medicine, University of Zagreb, Šalata 3, 10000 Zagreb, Croatia; 3 Faculty of Medicine Osijek, Josip Juraj Strossmayer University of Osijek, Josipa Huttlera 4, 31000 Osijek, Croatia; 4 School of Medicine, University of Split, Šoltanska 2, 21000 Split, Croatia

**Keywords:** inflammatory bowel disease, fractional exhaled nitric oxide testing, spirometry, pulmonary inflammation, child

## Abstract

**Highlights:**

**What are the main findings?**

- Children with active IBD have higher FeNO levels compared with healthy controls.
- FeNO levels positively correlate with CRP, while spirometric lung function remains normal across all IBD groups.

**What are the implications of the main findings?**

- Elevated FeNO may reflect underlying systemic inflammation in pediatric IBD, but its clinical utility as a marker of disease activity or relapse remains to be established.

**Abstract:**

**Objectives:** We aimed to assess fractional exhaled nitric oxide (FeNO) and spirometry in pediatric patients with inflammatory bowel disease (IBD) and relate these parameters to disease activity, duration, and current treatment. **Methods:** This prospective case–control study included 161 subjects: children with newly diagnosed, active IBD (N = 55), children in clinical remission (N = 53), and healthy controls (N = 53). FeNO was measured using a chemiluminescent analyzer, and pulmonary function was assessed by spirometry. **Results:** FeNO was higher in patients with IBD than in controls (*p* = 0.025) and positively correlated with CRP (ρ = 0.22; *p* = 0.027). Respiratory function measured by spirometry in children with IBD was preserved. No association was found between respiratory parameters, disease activity, and duration. The correlation between FeNO and aminosalicylate treatment was of borderline significance (ρ = 0.28; *p* = 0.052). **Conclusions:** Children with IBD, although having normal pulmonary function measured by spirometry, do have increased FeNO, which is positively correlated with CRP. FeNO reflects systemic inflammation, but its role as a clinical marker of disease activity or relapse remains uncertain.

## 1. Introduction

Inflammatory bowel disease (IBD) is a group of chronic inflammatory diseases that primarily affect the gastrointestinal tract. IBD occurs in two main clinical forms: Crohn’s disease (CD) and ulcerative colitis (UC). In addition to the digestive tract, various extraintestinal organ systems, including the respiratory tract, can be affected [1]. Respiratory involvement in adults with IBD encompasses airway disease (bronchitis, bronchiolitis, granulomatous bronchiolitis, and bronchiectasis); parenchymal disease (interstitial or organizing pneumonia, eosinophilic pneumonitis, and granulomatous lung disease); and, more rarely, thromboembolic events, pulmonary vasculitis, pleural disease, or enteric–pulmonary fistulae [2,3]. Many patients exhibit bronchial obstruction, airway hyperresponsiveness, small-airway dysfunction, pulmonary restriction, and impaired diffusion [4,5,6]. In addition, allergic asthma, abnormal pulmonary function tests, and positive prick test findings are more common in IBD patients than in control subjects [7,8].

Although respiratory manifestations in children and adolescents with IBD have been described only sporadically, mainly in case reports, their true prevalence is likely underestimated because pediatric patients often remain asymptomatic. Moreover, no recommendations exist for routine respiratory screening in pediatric IBD, despite reports of reduced lung function or radiological abnormalities even in asymptomatic children [9]. Previous studies in children with IBD that assessed fractional exhaled nitric oxide (FeNO) have yielded contradictory results [10,11,12,13,14,15,16]. By contrast, most adult data indicate that FeNO is associated with disease activity [17,18].

Therefore, we evaluated airway inflammation in children and adolescents with IBD by measuring FeNO levels and lung function using spirometry to determine whether these parameters correlated with disease activity, disease duration, and current treatment.

## 2. Materials and Methods

### 2.1. Study Design

This prospective case–control study included three groups of prospectively enrolled participants: children with newly diagnosed, active IBD before or immediately after starting treatment (Group A), children with IBD in clinical remission enrolled during follow-up visits (Group B), and healthy controls (Group C). Individuals with a history of atopy were not excluded from the control group, as excluding them would have introduced an imbalance between the groups. IBD was diagnosed according to the revised Porto criteria for IBD diagnosis in children and adolescents [19]. Eosinophilic gastrointestinal disease was histopathologically excluded in all patients. Clinical remission was defined by the Pediatric Crohn’s Disease Activity Index (PCDAI) or the Pediatric Ulcerative Colitis Activity Index (PUCAI) as less than or equal to 10 points [20,21].

The exclusion criteria were body mass index (BMI) or blood pressure at or above the 95th percentile for sex and age, antihypertensive treatment, dyslipidemia, diabetes mellitus, cardiovascular disease, or acute illness.

All patients underwent physical examination, and their weight and height were measured. C-reactive protein (CRP), erythrocyte sedimentation rate (ESR), hematocrit, and albumin levels were measured in the children with IBD. Eosinophil counts were recorded for all participants. The FeNO parameter was measured at an exhaled airflow rate of 50 mL/s using a chemiluminescence analyzer (Hypair FeNO, Medisoft, Sorinnes, Belgium). The children were not permitted to eat or drink for one hour before the measurements, as recommended. Measurements were repeated until adequate cooperation was achieved or if unusual values were obtained. The same measurement protocol was applied to all participants. FeNO values were analyzed according to the American Thoracic Society guidelines [22].

Lung function was determined by spirometry using a Spirolab MIR A23-0J spirometer (Medical International Research, Roma, Italy). The measured parameters were forced vital capacity (FVC), forced expiratory volume in the first second (FEV1), forced expiratory flow in the middle half of the forced vital capacity (FEF25-75), and FEV1/FVC ratio [23]. The data are expressed as z-values for age, sex, and height according to reference equations. The test was repeated until adequate cooperation was achieved [24].

The primary outcome was FeNO in pediatric patients with IBD in relation to disease activity and duration.

The secondary outcomes included lung function in children with IBD, measured by spirometry, and the effect of current treatment on respiratory parameters.

### 2.2. Statistical Analysis

The minimum sample size of 159 for the analysis of variance (with 80% power to detect a mean effect size at α = 0.05) was determined via a priori power analysis using G*Power (version 3.1.5) for Windows.

Data distribution was assessed using the Shapiro–Wilk test. We used the chi-square test to evaluate differences between categorical variables and the Kruskal–Wallis test for continuous variables, as the data were not normally distributed. For pairwise post hoc comparisons, we used the Mann–Whitney U test with the Holm correction for multiple comparisons. Categorical variables are presented as counts and percentages, whereas continuous variables are reported as medians and interquartile ranges. Correlations between respiratory parameters and inflammatory markers, duration, and disease activity were determined using Spearman’s correlation coefficient. Similarly, we assessed the correlation between respiratory parameters and current specific treatments. Multivariable linear regression models were used to evaluate the association between respiratory parameters and current treatment.

The significance threshold was set at *p* < 0.05. The analysis was performed using the IBM SPSS Statistics, Version 25.0 (IBM Corp., Armonk, NY, USA).

## 3. Results

### 3.1. Demographic Data

We included 161 subjects in the study and divided them into three groups based on IBD activity. Group A comprised 55 newly diagnosed patients (66% males; age 14.4 years (IQR 11.9–16.3 years)); Group B consisted of 53 patients in remission (53% males; age 15.6 years (IQR 13.5–16.7 years)); and Group C comprised 53 healthy subjects (55% males; age 14.1 years (IQR 11.2–16.1 years)). There were no significant differences between the groups in sex (*p* = 0.356), age (*p* = 0.077), or BMI z-score (*p* = 0.371). Group A comprised 24 patients with CD, 26 children with UC, and five subjects with IBD unclassified (IBDU). Group B consisted of 35 children diagnosed with CD, 16 with UC, and two with IBDU.

The disease lasted 3.7 (IQR 2.0–12.1) months in Group A and 44.51 (IQR 26.9–66.2) months in Group B. The PCDAI and PUCAI indices were 25.3 (IQR 18.5–36.9) and 35.0 (IQR 23.8–61.3) in Group A and 0.0 (IQR 0.0–2.5) and 0.0 (IQR 0.0–1.9) in Group B, respectively. In Group A, the ESR was 30.0 mm/h (IQR 13.5–48.5), while the CRP value was 13.9 mg/L (4.1–53.1). In Group B, ESR was 10.5 mm/h (IQR 6.0–18.0), and CRP was 1.4 mg/L (IQR 0.5–3.6). Eosinophil count was 180.0/μL in Group A (IQR 100.0–280.0), 150.0/μL in Group B (IQR 90.0–255.0), and 165.0/μL (IQR 80.0–315.0) in Group C. There was no significant difference in eosinophil count between the groups (*p* = 0.525), even after excluding four participants on corticosteroids (*p* = 0.584).

Our patients had predominantly extensive involvement of the digestive tract, as shown in Table 1.

At the time of enrollment, 16 (29%) patients with active disease and 16 (30%) patients in remission of IBD had extraintestinal manifestations (13 had liver and biliary tract disease, 16 had joint involvement, 2 had skin lesions, and one girl with CD had a pulmonary extraintestinal manifestation—pneumonia in the middle right lobe).

Two girls with UC, aged 5 and 14 years, had respiratory presentations of hypersensitivity reactions to aminosalicylates.

Tobacco exposure (smoking and home exposure) was similar between patients with IBD and controls (smoking in Group A (n = 3) vs. B (n = 2) vs. C (n = 3), *p* = 0.886; home tobacco exposure in Group A (n = 30) vs. B (n = 31) vs. C (n = 25), *p* = 0.494).

At the time of enrollment, 22 (42%) patients in Group B received azathioprine, 21 (40%) were treated with systemic aminosalicylates, 18 (34%) received anti-TNFα drugs, 11 (21%) received methotrexate, 4 (8%) received corticosteroids, and 3 (6%) had exclusive enteral nutrition (EEN).

### 3.2. Primary Outcome

**Figure 1:**
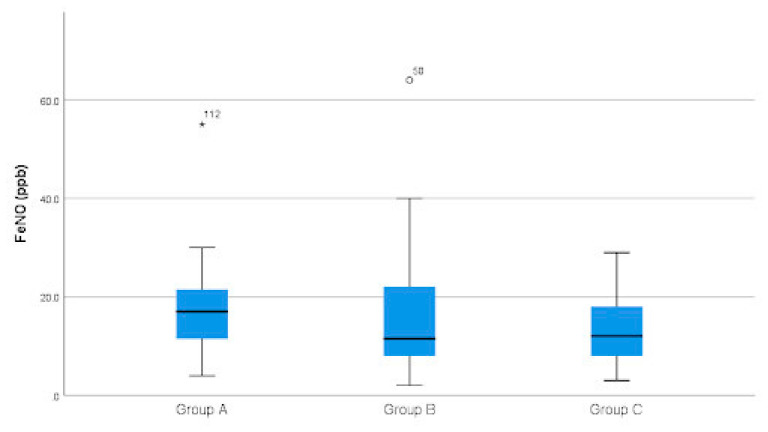
Fractional exhaled nitric oxide depending on the disease activity. Abbreviations: FeNO—fractional exhaled nitric oxide; Group A—patients with newly diagnosed active inflammatory bowel disease; Group B—patients with inflammatory bowel disease in remission; Group C—healthy subjects. °—mild outliers (values between 1.5 and 3 interquartile ranges from the box); *—extreme outliers (values more than 3 interquartile ranges from the box).

The FeNO values differed significantly among the three groups (*p* = 0.025). A significant difference was found between Groups A (17.0 ppb (IQR 11.0–22.0 ppb)) and C (12.0 ppb (IQR 7.5–18.0 ppb)) (*p* = 0.027), but not between Groups B (11.5 ppb (IQR 7.8–22.3 ppb)) and C (*p* = 0.532). The difference between Groups A and B was not significant (*p* = 0.104) (Figure 1). The sample size was not sufficient to reach corrected statistical significance when comparing FeNO in the three groups in Crohn’s disease (*p* = 0.049, Group A vs. C—*p* = 0.06 after Holm correction). In ulcerative colitis, FeNO did not seem to differ between the groups (*p* = 0.968).

FeNO values did not differ depending on the type of IBD (*p* = 0.130).

No significant difference was found between patients with previously diagnosed atopic disease (n = 37) and those without (n = 119) (*p* = 0.337). The difference was not statistically significant, even after stratifying the atopic participants into two groups: those with airway disease (n = 20) and those with non-airway atopic disease (*p* = 0.163). Moreover, FeNO did not correlate significantly with eosinophil count (ρ = 0.06, *p* = 0.505).

FeNO was positively correlated with CRP (ρ = 0.22, *p* = 0.027), whereas there was no significant correlation with the disease activity index (PCDAI ρ = 0.23, *p* = 0.092; PUCAI ρ = 0.11, *p* = 0.514), disease duration (ρ = 0.08, *p* = 0.294), or ESR (ρ = 0.11, *p* = 0.514).

### 3.3. Secondary Outcomes

Spirometric parameters FEV1, FVC, FEV1/FVC, and FEF25-75 did not differ between Groups A, B, and C.

Spirometry results were predominantly normal. Patients with IBD did not have a significantly higher number of pathological spirometries than those in the control group (11 (20.0%) in Group A vs. 11 (20.2%) in Group B vs. 7 (13.2%) in Group C, *p* = 0.669).

The measured spirometric parameters did not differ by IBD type.

We found no significant associations between lung function measures, disease activity, or disease duration. However, FeNO was positively correlated with the FEF25–75 parameter (ρ = 0.21, *p* = 0.023) and showed a positive trend with the FEV1/FVC parameter (ρ = 0.15, *p* = 0.067).

A borderline positive association was noted between FeNO and aminosalicylate treatment (ρ = 0.28, *p* = 0.052). In contrast, no significant correlations were observed between FeNO levels and any other medications used at the time of the study.

We noted a statistically significant correlation between current EEN treatment and the FEV1/FVC ratio (ρ = 0.286, *p* = 0.040). Nevertheless, only three patients received EEN at the time of study enrollment. 

In multivariable linear regression models, the measured respiratory parameters were not associated with current therapy. However, post hoc power analysis revealed that the number of patients in Group B was insufficient to achieve 80% power to detect a mean effect size (f2 = 0.15) at α = 0.05 (the minimum required sample size was 85). Consequently, we may have missed some statistically significant effects in this sample.

## 4. Discussion

The results of this study provide valuable insights into the respiratory aspects of IBD in children and adolescents. The combination of higher FeNO values in active IBD (particularly in CD), despite these patients’ slightly younger age, and their positive correlation with CRP suggests a potential link between systemic inflammation and respiratory involvement. This observation is consistent with findings in adults with IBD, in whom various pulmonary manifestations have been reported [2,4,5].

Our findings do not support eosinophilic inflammation as the underlying mechanism, as eosinophil counts were similar across all three groups, and FeNO values did not correlate with eosinophil counts.

Very few studies have measured the FeNO levels in children with IBD. Consistent with our results, Gut et al. found higher FeNO levels in patients with CD than in control subjects. The sample size of patients with UC was relatively small (N = 9). Two-thirds of the children with CD had active disease, as defined by the PCDAI. Although no association was found with the disease activity indices, a slight positive correlation was observed between FeNO and CRP levels [10]. Similarly, Huang et al. found slightly elevated FeNO levels (mean 25 ppb) in patients with CD (9 with active disease and 21 in remission) but with no correlation with PCDAI [11].

Based on the elevated FeNO levels in children with UC but not in those with CD, Perazynska et al. suggested that FeNO is a marker of airway involvement in pediatric patients with UC. Interestingly, patients with CD were predominantly in remission (21/25), whereas 14 of the 25 children with UC had active disease; therefore, disease activity, rather than the diagnosis itself, may be responsible for these results [12]. In addition, the same group of authors demonstrated the presence of airway inflammation in the absence of symptoms in children with IBD by measuring other inflammatory markers in exhaled air (interleukin 6 (IL-6), tumor necrosis factor-α (TNF-α), interleukin-1β (IL-1β), and interleukin 8 (IL-8)). No correlation was found between these parameters and disease activity or duration [25].

However, other studies in children with IBD have not reported elevated FeNO levels. Furlano et al. measured reduced lung diffusing capacity for carbon monoxide (DLCO), FEF25%, and FEF50% in children with CD and found no correlation with FeNO or disease activity. The median FeNO level was only 11.6 ppb [13]. Normal FeNO levels in children with UC and CD were reported by Yammine et al. [14] and Bak-Drabik et al. [15]. Livnat et al. found no difference in FeNO values between pediatric patients with CD and controls [16]. The subjects were mainly in remission across these studies, so this aligns with our finding that FeNO elevation was primarily observed in active disease. 

The significant limitations of these studies were the relatively small sample size and, most importantly, the inclusion of patients with active disease and those in remission in the same group. Our study sought to address these limitations by enrolling a large number of participants, stratifying patients by disease activity, and including an equal number of healthy children. Consistent with this, our data showed higher FeNO levels in children with active disease than in those in remission and in healthy controls. 

Despite airway inflammation, our findings indicate that spirometry-based lung function remains well preserved in children and adolescents. However, we observed a significant correlation between FeNO and FEF25–75, and a positive trend with the FEV1/FVC z-score, suggesting possible functional changes associated with respiratory inflammation. 

Previous studies in children with IBD have reported highly inconsistent results regarding lung function assessed by spirometry. 

Furlano et al. found a slight reduction in flow at the level of the small airways in patients with CD. These changes did not persist in follow-up spirometric measurements after a median of 34 months [13]. Amrousy et al. reported a significant decrease in FEV1, FVC, and FEF25-75 during active disease and a negative correlation between these parameters and disease activity [26]. Bak Drabik et al. reported a decline in all spirometric parameters in children with IBD, most of whom were in remission [15].

However, consistent with our results, Welsh et al. reported normal spirometric findings in children with IBD. They observed no significant association between spirometry parameters and disease duration or hospitalizations [27]. Several studies have reported comparable results [10,14].

Differences in spirometry results can be attributed to heterogeneity across studies in terms of methodology, disease activity, treatment, and small sample sizes. At the same time, changes in functional lung tests observed in the study by Furlano et al. did not persist beyond a median follow-up of 34 months, suggesting that lung function in children with IBD varies over time [13].

While lung manifestations are more common in adult patients with UC, most reported cases in children and adolescents involve CD [28]. Our only patient with an overt respiratory manifestation of IBD—right-sided middle lobe pneumonia—also had CD.

The results of our study contribute to the understanding that subtle respiratory changes may be detectable in the early stages of IBD even in the absence of spirometry abnormalities and overt clinical symptoms. In addition, the study results underline the need for a longitudinal follow-up to detect respiratory changes over time. Understanding whether children with elevated FeNO levels are at increased risk of developing clinically apparent respiratory manifestations or relapse could have significant implications for patient management.

Distinguishing respiratory manifestations of IBD from treatment-related symptoms is often challenging. Although there have been dozens of reports on aminosalicylate-induced lung disease in adults, there are only a few reports on the adverse effects of mesalazine in children [29,30,31,32]. Two girls in our cohort developed aminosalicylate-induced lung disease at the ages of 5 and 14 years. The pathophysiological mechanisms underlying aminosalicylate-induced lung injury remain unclear. Immune-mediated alveolitis, resulting from lymphocyte stimulation, dose-dependent lung damage, or the direct toxic effect of aminosalicylates, and oxidative damage to the lung epithelium have been proposed [33]. 

Regarding treatment, the only association observed was between FeNO values and aminosalicylate therapy, which was statistically significant at the borderline level. However, this observation is noteworthy, given the reported hypersensitivity reactions to aminosalicylates, and highlights the need for careful monitoring and consideration of alternative treatments when respiratory symptoms occur. 

The main drawbacks of this study arise from its cross-sectional design. A further limitation is the presence of multiple factors that may influence FeNO values independently of systemic inflammation in IBD. We attempted to control for several known confounders, including atopy, recent respiratory infection, anthropometric characteristics, age, sex, smoking exposure, pre-measurement fasting, and technical aspects of the procedure. However, additional unmeasured factors—such as habitual dietary nitrate intake, recent physical activity, or other lifestyle-related influences—may also have contributed to variability in FeNO values [22]. However, the large sample size and strict diagnostic criteria have enhanced the data’s validity in areas where prior research has been limited.

In conclusion, children with IBD in our study had preserved lung function, as measured by spirometry, but exhibited elevated FeNO levels that correlated positively with CRP. Therefore, FeNO reflects systemic inflammation, but its role as a clinical marker of disease activity or relapse remains uncertain.

## Figures and Tables

**Table 1:** Intestinal involvement in patients with inflammatory bowel disease according to the Paris classification.

	Group A, n (%)	Group B, n (%)
Ulcerative colitis		
E1	1 (4)	0 (0)
E2	2 (8)	2 (13)
E3	5 (19)	2 (13)
E4	16 (62)	11 (69)
Partial colonoscopy	2 (8)	1 (6)
Crohn’s disease		
L1	3 (13)	2 (6)
L2	2 (8)	3 (8)
L3	19 (79)	30 (86)
L4a present	9 (38)	16 (46)

Group A—patients with newly diagnosed active inflammatory bowel disease; Group B—patients with inflammatory bowel disease in remission.

## Data Availability

Data are available upon request from the authors.

## Abbreviations

The following abbreviations are used in this manuscript:

IBD	Inflammatory bowel disease
CD	Crohn’s disease
UC	Ulcerative colitis
FeNO	Fractional exhaled nitric oxide
PCDAI	Pediatric Crohn’s Disease Activity Index
PUCAI	Pediatric Ulcerative Colitis Activity Index
BMI	Body mass index
CRP	C-reactive protein
ESR	Erythrocyte sedimentation rate
FVC	Forced vital capacity
FEV1	Forced expiratory volume in the first second
FEF25–75	Forced expiratory flow between 25% and 75% of FVC
IBDU	Inflammatory bowel disease unclassified
EEN	Exclusive enteral nutrition
IL-6	Interleukin-6
TNF-α	Tumor necrosis factor-α
IL-1β	Interleukin-1β
IL-8	Interleukin-8
